# The impact and origin of copy number variations in the *Oryza* species

**DOI:** 10.1186/s12864-016-2589-2

**Published:** 2016-03-29

**Authors:** Zetao Bai, Jinfeng Chen, Yi Liao, Meijiao Wang, Rong Liu, Song Ge, Rod A. Wing, Mingsheng Chen

**Affiliations:** State Key Laboratory of Plant Genomics, Institute of Genetics and Developmental Biology, Chinese Academy of Sciences, Beijing, 100101 China; State Key Laboratory of Systematic and Evolutionary Botany, Institute of Botany, Chinese Academy of Sciences, Beijing, 100093 China; Arizona Genomics Institute, School of Plant Science, University of Arizona, Tucson, AZ 85721 USA

**Keywords:** *Oryza* species, Copy number variation (CNV), NGS-based survey, CNV genes, Mutation mechanisms

## Abstract

**Background:**

Copy number variation (CNV), a complex genomic rearrangement, has been extensively studied in humans and other organisms. In plants, CNVs of several genes were found to be responsible for various important traits; however, the cause and consequence of CNVs remains largely unknown. Recently released next-generation sequencing (NGS) data provide an opportunity for a genome-wide study of CNVs in rice.

**Results:**

Here, by an NGS-based approach, we generated a CNV map comprising 9,196 deletions compared to the reference genome ‘Nipponbare’. Using *Oryza glaberrima* as the outgroup, 80 % of the CNV events turned out to be insertions in Nipponbare. There were 2,806 annotated genes affected by these CNV events. We experimentally validated 28 functional CNV genes including *OsMADS56*, *BPH14*, *OsDCL2b* and *OsMADS30*, implying that CNVs might have contributed to phenotypic variations in rice. Most CNV genes were found to be located in non-co-linear positions by comparison to *O. glaberrima.* One of the origins of these non-co-linear genes was genomic duplications caused by transposon activity or double-strand break repair. Comprehensive analysis of mutation mechanisms suggested an abundance of CNVs formed by non-homologous end-joining and mobile element insertion.

**Conclusions:**

This study showed the impact and origin of copy number variations in rice on a genomic scale.

**Electronic supplementary material:**

The online version of this article (doi:10.1186/s12864-016-2589-2) contains supplementary material, which is available to authorized users.

## Background

One of the most important findings of comparing related genomes was the widespread copy number variations (CNVs) in eukaryotic genomes. CNVs, also called unbalanced structural variations, include deletions, insertions, and duplications of ≥ 50 bp in size, which can change gene structure and dosage, and modify gene regulation [1, 2]. However, among all the forms of genetic variations present in a genome, CNV is one of the most difficult to genotype and elucidate their evolutionary consequences [3]. Since a larger fraction of the genome were affected by CNVs other than single nucleotide polymorphisms, CNVs are responsible for more heritable differences between individuals, implying their important roles in phenotypic variations [4, 5]. CNVs are likely to have significant functional impacts on genes and may explain some phenotypic variations not captured by SNP-based studies [6]. Many detailed studies have been performed to interpret the relationship between CNVs and phenotypic variations in mammalian genomes [7–10], *Drosophila* [11–14], and domestic animals [15–19]. In humans, many CNVs have been linked to various diseases and traits [3] and most of them can lead to genetic and phenotypic difference between individuals and populations [5]. Furthermore, ancient CNVs that differ between human and non-human primates have led to species-specific phenotypes [20–22].

In plants, there are growing evidences indicating that genes affected by CNVs are associated with important traits. For example, CNVs at the *Rhg1* locus can mediate resistance to soybean cyst nematode [23]; CNV in a transporter gene (*MATE*1) of maize was found to be the genetic basis for aluminum tolerance [24]. In barley, increased copy number of a boron transporter gene (*Bot1*) conferred tolerance to boron-toxicity [25]. In rice (*Oryza sativa*), a deletion in *qPE9-1* is associated with panicle erectness [26], a deletion of the *qSW5* gene caused the increase in grain size [27], and a duplication of *GL7* locus contributed to grain size diversity [28]. However, the exploration of the extent and role of CNVs in plants is still just beginning. Several recent studies have provided a first glimpse of plant CNVs on a genomic scale. In maize, CNVs and Presence/Absence Variations were pervasive in maize inbreed lines [29, 30], and most of them were enriched at loci associated with important traits [31]. Combined with other genome analyses in soybean [32, 33], rice [34–36], *Arabidopsis* [37–39], sorghum [40], wheat [41], and barley [42], these results showed that genes affected by CNVs were significantly enriched in defense responses, and responses to stresses.

CNVs have emerged as a consequence of errors in DNA recombination, replication, and repair-associated processes [3, 43]. The detailed understanding of CNV mutation mechanisms in eukaryotes is mainly based on DNA double-strand break (DSB) repair studies in bacteria, yeast, and other mammalian somatic cells [44–46]. In general, there are two pathways for DSB repair: (1) non-homologous recombination (NHR), also named illegitimate recombination, which includes non-homologous end joining (NHEJ) and microhomology-mediated end joining (MMEJ), and can be independent of sequence homology, or only requiring microhomology patches of 1–10 bp; (2) homology-based repair including non-allelic homologous recombination (NAHR), which requires extensive regions of sequence homology (usually several hundred base pairs) [45, 46]. By examining the sequence context of CNV regions and breakpoints, other mutational processes have also been characterized, including mobile element insertion (MEI) and shrinking or expansion of variable number of tandem repeats (VNTRs) [47] mediated by misalignment of repetitive DNA sequences [44].

The genus *Oryza* consists of 24 species including the Asian cultivated rice [48]. Because of its diversity of species, well-characterized phylogeny, and rich genomic resources, the genus *Oryza* became an ideal model for studies of genome evolution [49]. Recently, the availability of genome sequencing data for several *Oryza* species provided an opportunity to explore structural variations and mechanisms underlying *Oryza* genome evolution [50–52]. Several studies have demonstrated the prevalence of CNVs in the *Oryza* species [34–36]; however, detailed analyses of the impact and origin of CNVs have not been performed. The identification of precise CNV sequences is a crucial prerequisite for detailed CNV characterization and functional analysis [47]. Compared to comparative genomic hybridization (CGH)-based survey, next-generation sequencing (NGS)-based method have enabled CNV mapping at single-nucleotide resolution [53–58]. In the present study, we generated a CNV map at single-nucleotide resolution using NGS-based approach for 50 rice accessions [36]. The high-resolution CNV map enabled us to elucidate the functional impacts and mutational mechanisms of CNVs in the *Oryza* species.

## Results

### CNV discovery

**Fig. 1:**
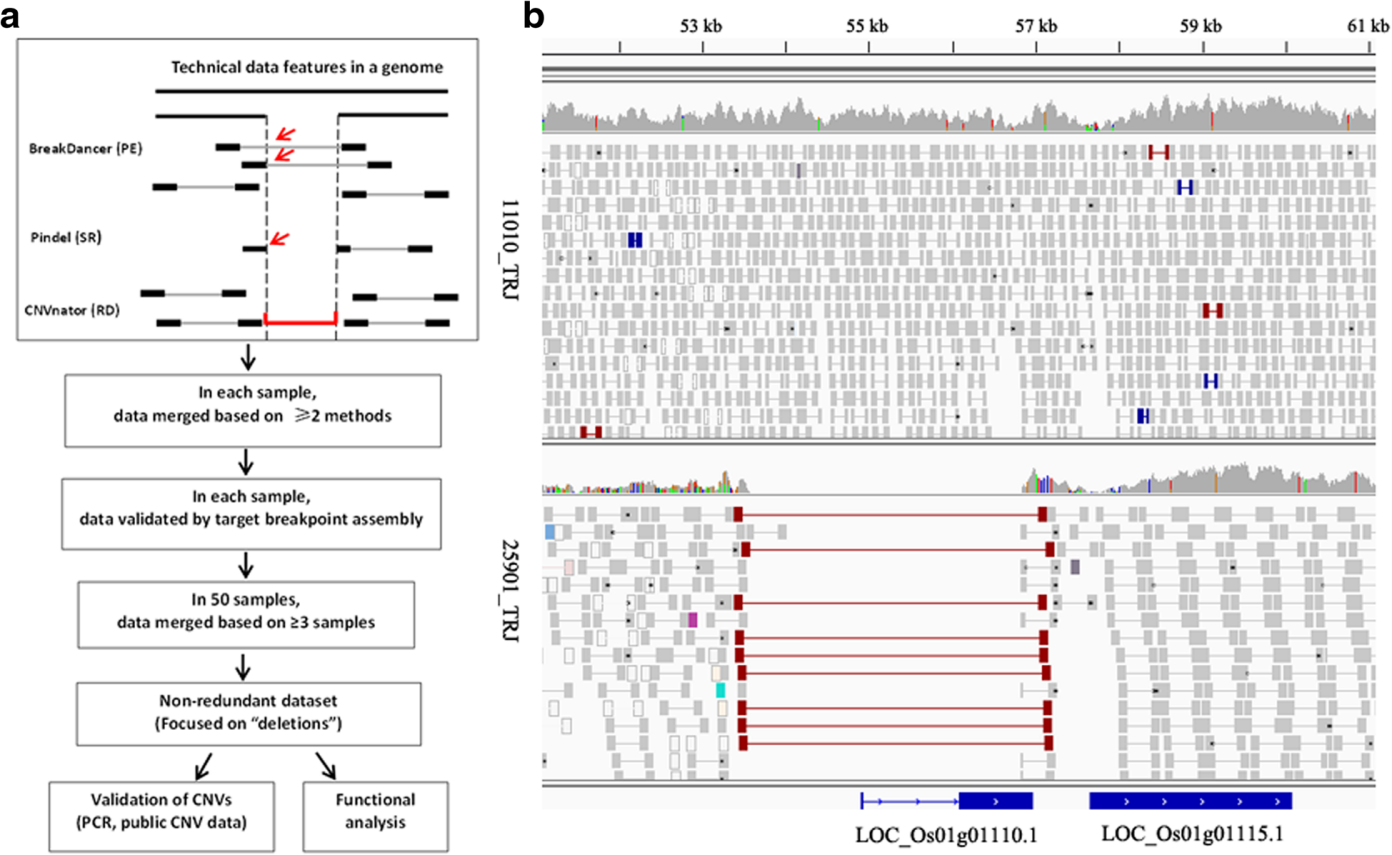
Copy number variations discovered using NGS-based survey. **a** The pipeline of CNV discovery and validation. **b** Example of a CNV supported by abnormal pair-end reads (red) and reads-depth (dark grey peak). Light grey boxes represent pair-end reads. 25901_TRJ and 11010_TRJ are two rice accessions with or without the CNV, respectively

We applied three complementary approaches to identify CNVs in the *Oryza* species: (1) paired-end (PE) mapping based on analysis of abnormally mapping reads of size-selected clone ends [53, 54, 59]; (2) split-read (SR) analysis, which examines gapped alignments of DNA reads [2, 60]; (3) read-depth (RD) analysis, which detects CNVs by analyzing the read depth-of-coverage [55, 58, 61, 62]. To obtain a full range of high-confidence CNVs detectable by these complementary sequence features (PE, RD, and SR), we integrated the results from three CNV discovery tools: BreakDancer [63], CNVnator [61], and Pindel [60]. This approach is depicted in Fig. 1.

We focused initially on deletions compared to the reference genome Nipponbare. In total, we detected 9,196 deletions with sizes ranging from 62 to 654,630 bp (mean 4,166 bp and median 1,118 bp). Most of these deletions (9,015 of 9,196; 98 %) were inferred breakpoints at single nucleotide resolution, representing a genome-wide, base-pair resolution CNV catalog in the *Oryza* species (Additional file 1: Table S1). The CNVs were defined as deletions relative to the reference genome. To determine whether these CNVs are deletions or insertions in an evolutionary context, we introduced *Oryza glaberrima* as the outgroup [51]. By comparing to the orthologous regions in *O. glaberrima*, we re-defined the variation types of this CNV dataset: among 8,929 deletion events, 7,400 (80 %) are actually insertions, 1,526 (17 %) are *bona fide* deletions, and 270 (3 %) were not defined due to sequence gaps in *O. glaberrima* (Additional file 1: Table S1).

### Extensive validation of CNVs

To assess the quality of this CNV dataset, we performed PCR validation for 90 candidate loci. We performed PCR experiments in five rice accessions, and 76.7 % (69/90) of the CNV events were verified (Additional file 1: Table S1). We also compared the dataset with recently reported CNV data by read-depth based method only [36]. These two datasets were overlapping for 68 % (6,210 events) (Additional file 1: Table S1).

Next, we assessed the data by comparison with a microarray-based study in *japonica* and *indica* subspecies [34] and a BAC-based report between rice and three of its closest relatives [35]. Only 80 events were overlapped with the microarray data and three with the BAC data (Additional file 1: Table S1). A possible explanation for this small overlap is that different size ranges were detected by different methods. While previously reported CNVs were focused on large-sized events, this data are mainly composed of intermediate-sized CNVs, with 87 % (7,986/9,196) smaller than 10 kbp.

### Impact of CNVs on genes and gene function

The single nucleotide resolution of the CNV map enabled us to evaluate the functional consequences of CNVs on genes and gene function. In total, 2,806 genes were affected by 2,879 CNVs, and the coding regions of 1,675 genes were disrupted by CNVs, causing 558 partial gene deletions and 1,117 full gene deletions (Table 1). We next evaluated the population distribution for 720 CNV events which affect 1,117 full genes. Nearly 81.7 % CNVs were shared by both cultivated and wild rice, whereas 0.8 % was observed in wild rice, and 17.5 % was present in cultivated rice. The identification of fewer wild-specific CNVs could be a consequence of the inclusion of fewer wild rice lines (10) in this study, and sequence reads from wild accessions that may could not be mapped to the reference genome. We further assessed the distribution of CNVs in subpopulations involving the *O. sativa* subspecies *japonica* (24) and *indica* (13). The proportion of CNVs detected only in *indica* was 0.7 %, and 3.9 % in *japonica*. The remaining 12.9 % was shared by both of them (Additional file 2: Table S2; Additional file 3: Table S3). The majority of CNVs were shared by cultivated and wild rice or *indica* and *japonica*, suggesting that most of these CNVs were derived from the same gene pool. The Gene Ontology (GO) analysis of 1,675 CNV genes suggested that they were significantly enriched in functional categories involved in interactions with the environment, including apoptotic processes, responses to stresses, hypersensitive responses and others (Additional file 4: Figure S1; Additional file 5: Table S4). However, when we focused on 1,117 full genes affected by CNV, their function are enriched in apoptotic process (Additional file 5: Table S4).

**Table 1:** Overview of the CNV dataset in 50 rice accessions

*Oryza* species	CNV Total	Gene overlap					
		CNV Total	Genes Total	Full genes	Partial CDS	UTR	Intron
50 rice accessions	9196	2879	2806	1117	558	470	661

**Fig. 2:**
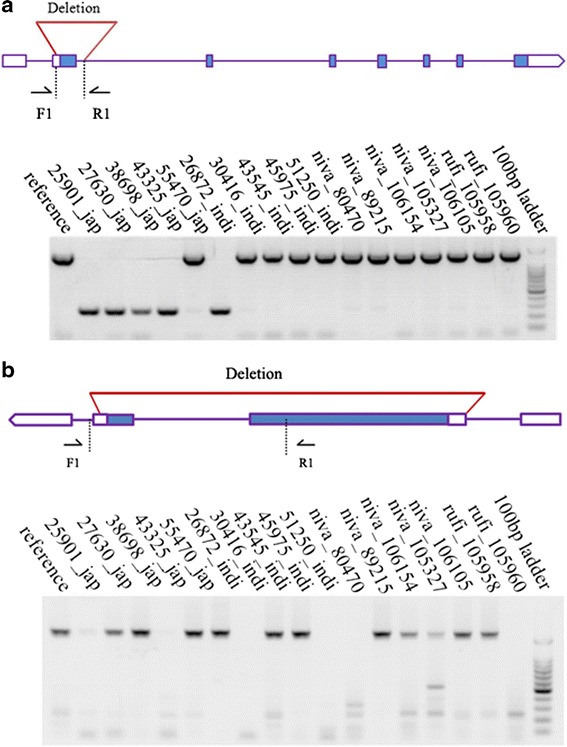
PCR validation of CNVs in *OsMADS56* and *BPH14*. **a** Schematic gene structure and CNV position of *OsMADS56.* F1: 5’-TCGTCGAGCCATTTCGGG-3’ and R1: 5’-CCATTTGTAGTCTCGCACGCTC-3’. **b** Schematic gene structure and CNV position of *BPH14*. F1: 5’-ATCAGGGTCCACCAGCGAGA-3’ and R1: 5’-GCCAGCCAGCAAAATATCTTTA-3’. PCR validations were performed in 18 rice accessions. Blue boxes stand for CDS; white boxes depict UTR; inverted triangles indicate CNV positions

We further identified and validated a number of previously undescribed functional genes interrupted by CNVs, including five genes in the coding regions and 23 in non-coding regions (Additional file 6: Table S5). *OsMADS56* (LOC_Os10g39130), which consists of eight exons, encodes a typical MIKC-type MADS-box protein. Overexpression of *OsMADS56* resulted in delayed flowering under long day condition, while a loss-of-function mutation had no alterations in timing of flowering [64, 65]. A CNV encompassing the first exon of *OsMADS56* resulted in partial deletion of the MADS-box domain (Fig. 2a).

*BPH14* (LOC_Os03g63150) confers resistance to brown planthopper in rice. It encodes a coiled-coil, nucleotide-binding, and leucine-rich repeat (CC-NB-LRR) protein. The sequence variations in LRR domain are responsible for the function in insect resistance [64, 66]. A CNV spanning the entire *BPH14* gene was detected and validated by PCR experiments (Fig. 2b).

**Fig. 3:**
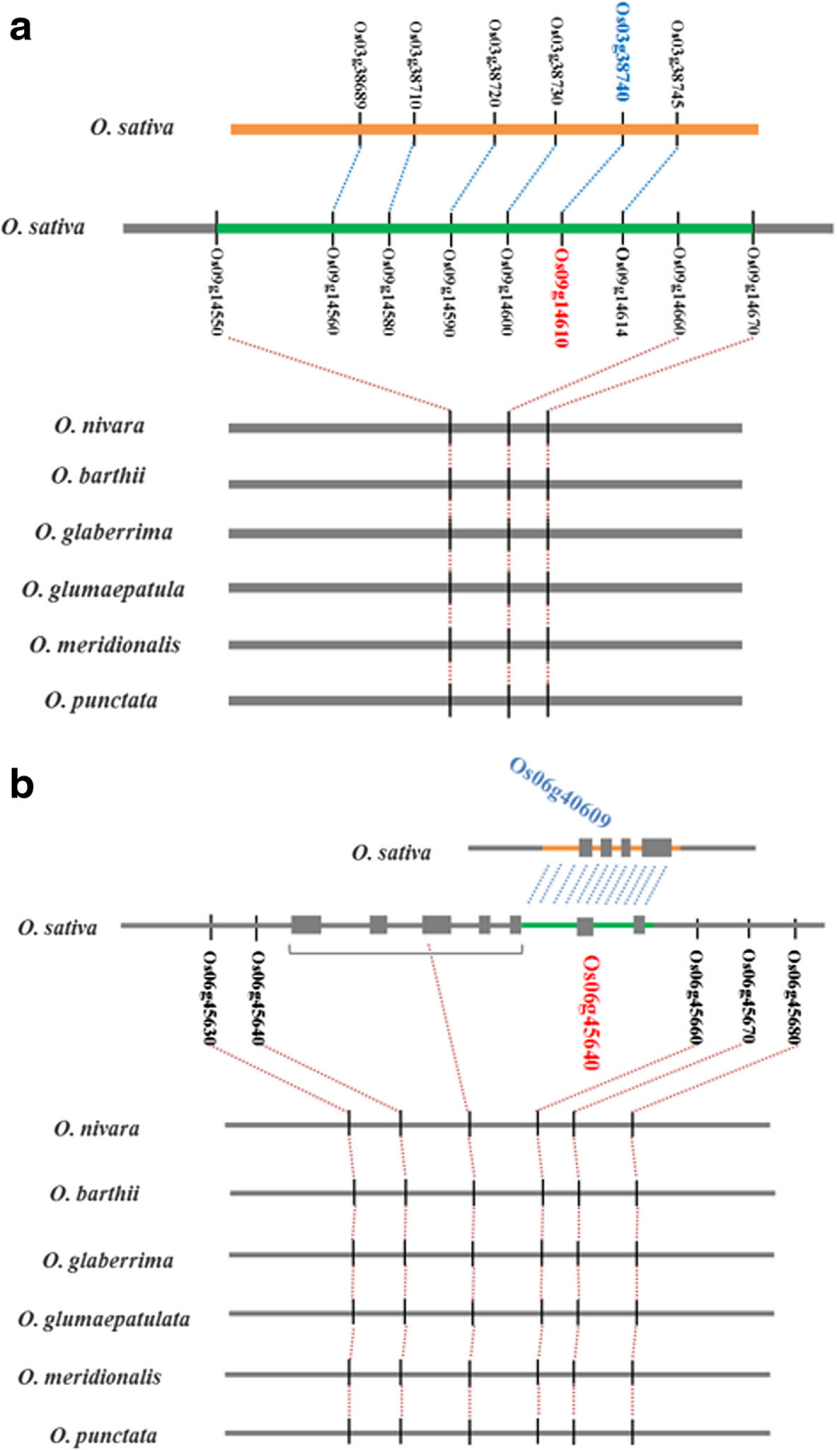
PCR validation of CNVs in *OsDCL2b* and *OsMADS30*. **a** The CNV enclosing *OsDCL2b* was not present in orthologous regions of *O. nivara*, *O. barthii*, *O. glaberrima*, *O. glumaepatula*, *O. meridionalis*, and *O. punctata. OsDCL2b* (red) was duplicated from *OsDCL2a* (blue) as part of a large segmental duplication. **b** The CNV covering the last two exons of *OsMADS30* was present in *O. sativa*, but not in *O. nivara*, *O. barthii*, *O. glaberrima*, *O. glumaepatula*, *O. meridionalis*, and *O. punctata*. This fragment was duplicated from a genomic region enclosing Os06g40609 on the same chromosome. Gray horizontal lines, orthologous regions of the *Oryza* species; green lines, CNVs; yellow lines, homologous regions of CNV; gray vertical lines, genes; gray boxes; exons. Orthologous genes are connected by red lines. Homologous genes are connected by blue lines

*OsDCL2b* (LOC_Os09g14610), a Dicer-like gene, participates in the regulation of gene silencing at the post-transcriptional level by RNA interference [67]. A large CNV (65 kb) enclosing *OsDCL2b* was identified. Local alignments showed that this fragment was present in the *O. sativa*, but absent in *Oryza nivara*, *Oryza barthii*, *Oryza glumaepatula*, *Oryza meridionalis* and *Oryza punctata*, implying it was actually an insertion in *O. sativa*. Further analysis verified that *OsDCL2b* is a duplication of *OsDCL2a* as part of a large segmental duplication from chromosome 3 to 9 (Fig. 3a), which is consistent with a previous report [68].

*OsMADS30* (LOC_Os06g45650) encodes MIKC-type MADS-box protein, and participates in the response to dehydration and salt stress [69]. A CNV spanning the last two exons of *OsMADS30* was detected. Comparative sequence analysis demonstrated that this CNV was only present in *O. sativa*, indicating that it is an evolutionarily recent insertion. This fragment was duplicated from a genomic region enclosing LOC_Os06g40609 on the same chromosome (Fig. 3b). Therefore, *OsMADS30* was a new gene formed by gene fusion in *O. sativa*.

### Formation mechanisms of non-co-linear CNV genes

**Fig. 4:**
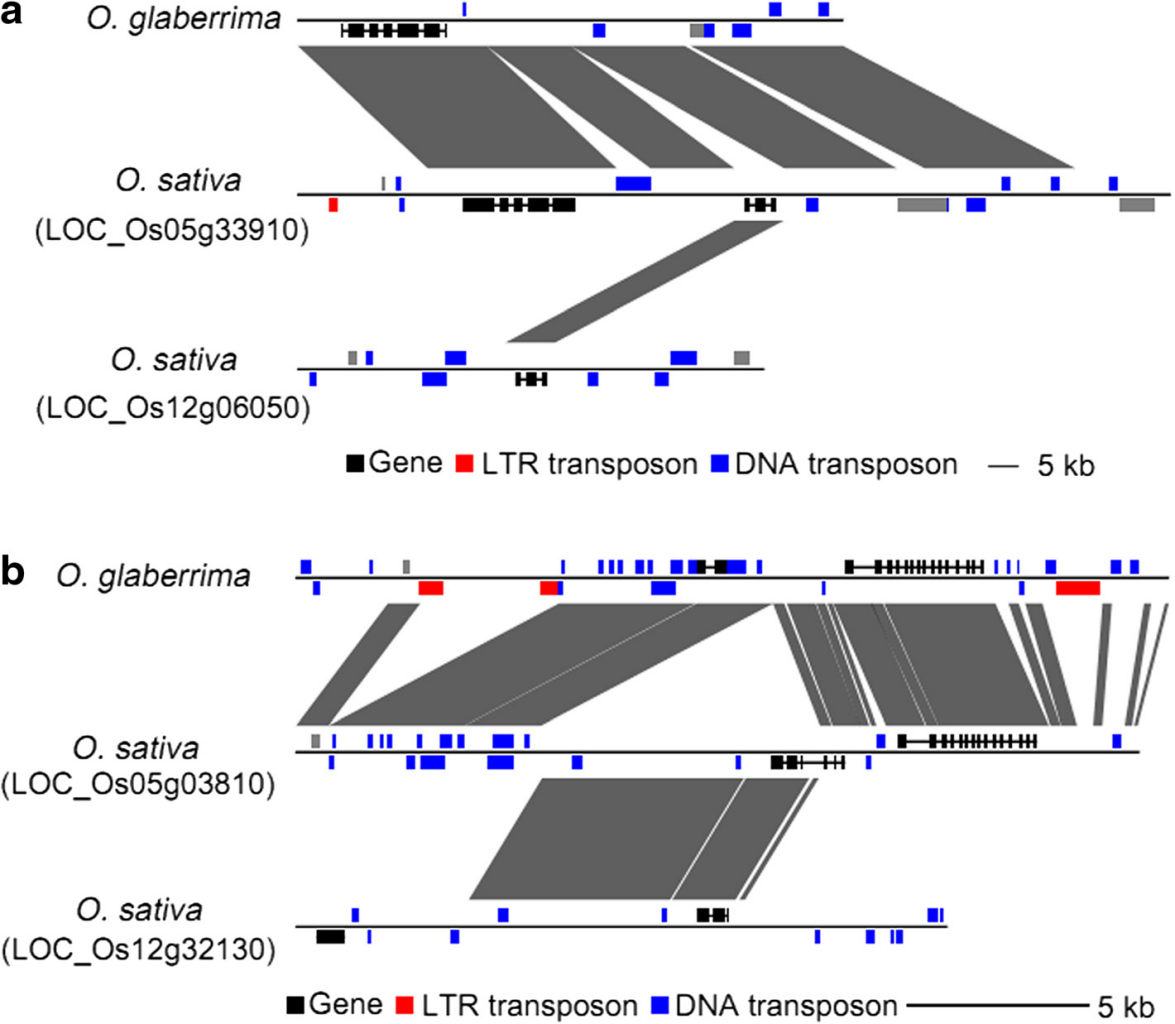
Sequence analysis of the origin of non-co-linear CNV genes. The regions containing non-co-linear CNV genes were compared with orthologous regions in *O. glaberrima*. **a** The non-co-linear CNV gene LOC_Os05g33910 was a duplicate of LOC_Os12g06050. **b** A CNV containing LOC_Os05g03810 was duplicated from a segment spanning LOC_Os12g32130

Many CNV genes are actually insertions in Nipponbare, thus form non-co-linear genes in the *Oryza* species. Among 697 conserved genes whose coding regions were affected by CNVs, 287 of them are non-co-linear; majority of them (260/287) have a homolog in the Nipponbare genome with sequence identity ranging from 80 % to 100 % (Additional file 7: Table S6), implying that these non-co-linear genes were possibly duplicated from other places in the genome (Fig. 4).

Although studies have been conducted to reveal mechanisms of non-co-linear genes in *Drosophila* [70, 71] and plants [72–75], ancient gene transposition provided insufficient sources of clues due to sequence decay by random mutations. Comparison of more closely related species will increase the power of evolutionary inference. In this study, the divergence time between *O. sativa* and *O. glaberrima* is less than 2 million years [76]. A recent duplication event would leave an ancestral copy in the original syntenic position. By comparative sequence analysis, diagnostic motifs such as target site duplications and precise borders can be identified, and thereby, mechanisms underlying the formation of non-co-linear CNV genes can be inferred more precisely.

**Fig. 5:**
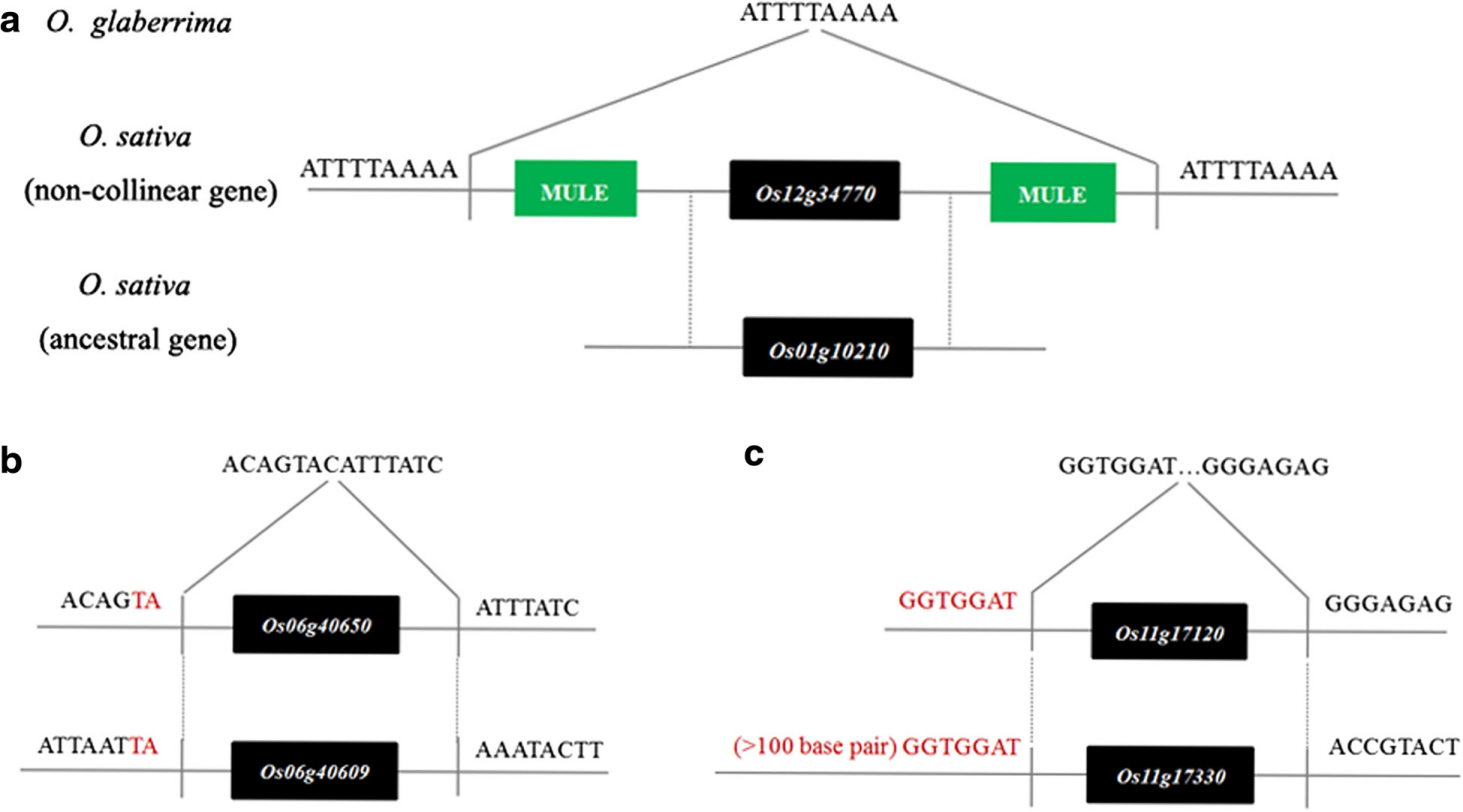
Formation mechanisms of non-co-linear CNV genes inferred by signature of breakpoint. **a** By comparison to the ancestral gene *Os01g10210*, *Mutator*-like elements flanked both sides of *Os12g34770,* implying that this duplication event was possibly mediated by TE activity. **b** Micro-homology at breakpoints between *Os06g40650* and *Os06g40609* indicated this duplication was mediated by NHEJ (non-homologous end joining) during DSB repair process. **c** High homology at breakpoint between *Os11g17120* and *Os11g17330* indicated the role of NAHR (non-allelic homologous recombination). Black boxes, genes; green boxes, *Mutator*-like elements; red characters, homologous sequences between the non-co-linear gene and its ancestral copy

Here, we focused on high-scoring homologous gene pairs which are at least 90 % identical. The non-co-linear genes (27) were aligned to their respective ancestral copies, and the mechanisms underlying their formation were deduced by examining signatures of breakpoints. Transposable elements flanked both sides of the non-co-linear genes, implying that these duplication events were possibly mediated by TE activity (Fig. 5a); micro-homology or no homology at breakpoints between the non-co-linear CNV gene and its ancestral copy indicate that NHEJ (non-homologous end joining) appears to be at play during DSB repair process (Fig. 5b); high homology at breakpoints support the role of NAHR (non-allelic homologous recombination) (Fig. 5c). In total, 12 cases were formed by TEs, 14 cases by NHEJ, and 1 case by NAHR (Additional file 8: Table S7).

**Fig. 6:**
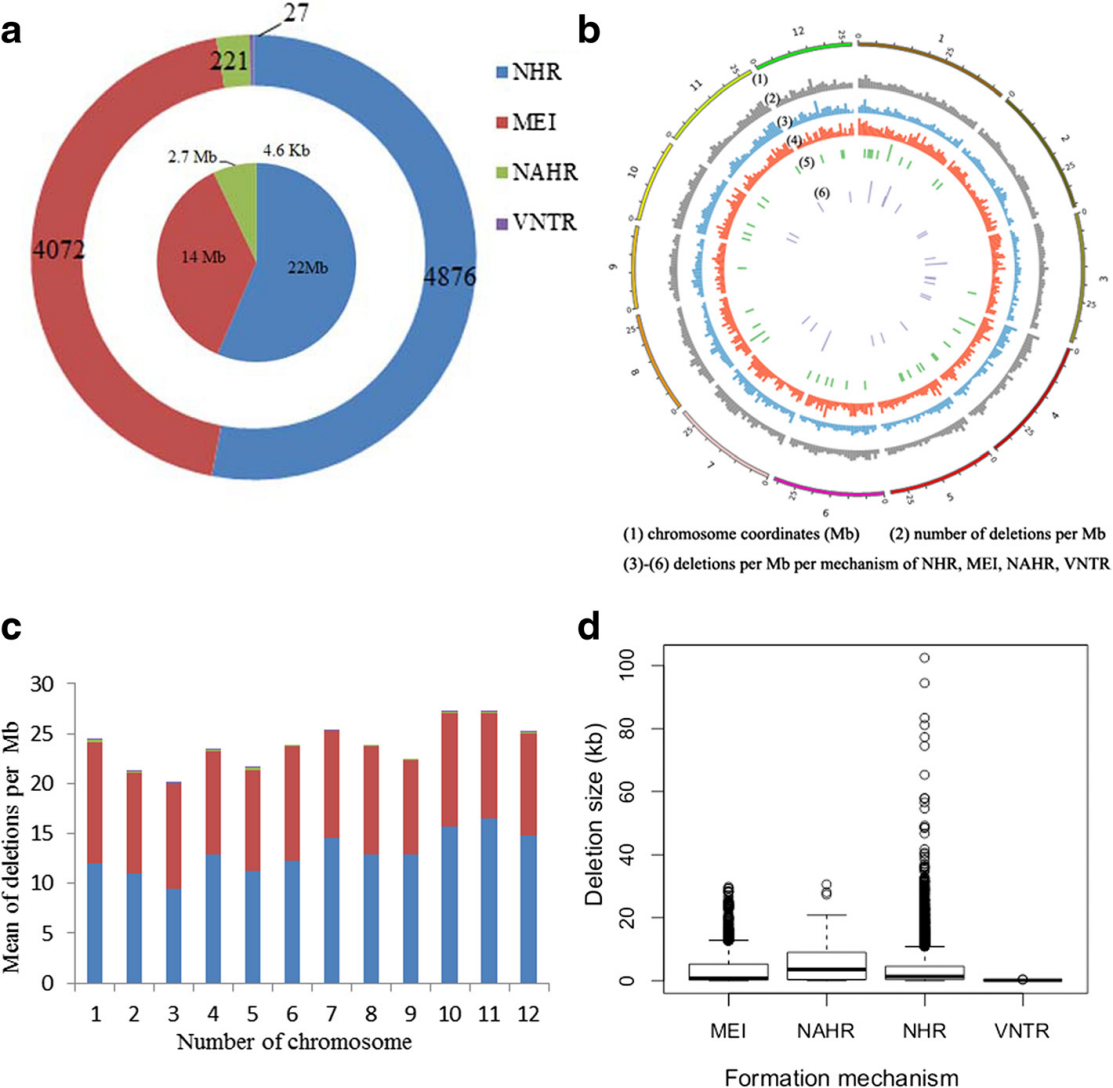
CNV formation mechanisms acted on the rice genome. **a** Distribution of different CNV formation mechanisms (9015 CNVs). Outer circle represents number of CNVs per mechanism. Inner circle represents cumulative genomic size of these events. **b**, **c** Spatial distribution of CNVs formed by different formation mechanisms on the 12 chromosomes. The color represents different mechanisms as in (**a**). **d** CNV size comparison formed by different mechanisms

We next sought to determine the formation mechanisms of the entire CNV dataset. With the benefit of single base-pair resolution, the relative contribution of each mutational mechanism can be defined. We applied the BreakSeq pipeline, which scans specific diagnostic sequence signatures at breakpoint junctions to infer the formation mechanism of each CNV [47]. Eventually, 52.98 % (4,872) and 44.28 % (4,072) were found to be formed by NHR (non-homologous recombination) and MEI (mobile element insertion), respectively, and 0.48 % (44) and 0.29 % (27) by NAHR and VNTR, respectively. The remaining 1.97 % (181) was too ambiguous to be defined (Figs. 6a-c; Additional file 9: Table S8). By relating the formation mechanisms to CNV size, we observed a broad size range in MEI, NAHR, and NHR, whereas there was a relatively small range of CNV sizes in VNTR (Fig. 6d).

## Discussion

Although structural variations >100 bp in length have been identified for this resequencing data, the breakpoint cannot be precisely determined due to limitation of the read-depth method [77]. In this study, we re-generated the variation by three complementary short read-based surveys, which can improve the confidence of CNV events and the precision of CNV boundaries. Based on this CNV map, we emphasized the impact and origin of this type of genomic variation.

Most CNVs are actually insertions in *O. sativa*, which implies that insertions are predominant in the rice genome evolution. A recently published paper also showed that natural insertions in rice were commonly occurred [78]. These results are consistent with previous reports that the rice genome has experienced massive recent amplifications in the last two million years [50, 79].

In this study, we detected and validated 28 functional CNV genes. The coding regions of five genes were affected by CNVs, including *OsMADS56*, *BPH14*, *OsDCL2b, OsMADS30* and *OsWAKY8*. Because of their important functions, we envision that the variation in these genes may have functional consequences. However, genes identified, as with CNV genes reported previously, are all members of multigene families. The deletion or duplication of CNV genes can be genetically buffered. Therefore, genes affected by CNVs may contribute to quantitative rather than qualitative variations [29, 80–82].

CNV genes tend to locate in regions with low levels of conservation among species. Nearly 58 % (978/1,675) CNV genes are rice-specific; among the remaining 697 conserved CNV genes, 41 % are non-co-linear ones. The gene order in animal genomes has been conserved over millions of years, while co-linearity in plants genomes was dramatically disturbed [82, 83]. The number of co-linear genes decreases with increasing phylogenetic distances. Recent works indicated that many non-transposon genes and gene families are capable of moving in plants [72, 74]. One possible mechanism is DNA-based “copy and paste” mediated by transposons or recombination. Transposons can occasionally “capture” genic sequence fragments and move them to other locations in the genome, such as *Mutator* [84], *Helitron* [85, 86], and *LTR* retrotransposons [87]. An alternative mechanism of gene capture is the repair of DSB by NAHR, NHEJ or MMEJ. This study indicated that both transposon activity and recombination were involved in the formation of CNV genes in rice.

In this study, we were unable to provide the direct link between CNVs and phenotypes, which is rather challenging by using reverse genetic approaches. However, we believe that this CNV map will be of great value for future association studies by either eQTL (expression quantitative trait locus) or GWAS (genome-wide association study) to relate CNV genotypes to phenotypes [11, 12, 88, 89].

## Conclusions

By three complementary NGS-based methods, we performed genome-wide CNV detection based on published sequencing data of 50 rice accessions. The study demonstrated that 28 functional genes were disrupted by CNVs, and the main mechanisms of CNV formation in rice were NHR and MEI. We foresee that this CNV map will be of great value for studying genome evolution and phenotypic variation in the *Oryza* species.

## Methods

### Next-generation sequencing data, and reads mapping

The Illumina paired-end read sequencing data for 50 rice accessions were obtained from the published paper with accession number SRA023116 in NCBI Short Read Archive [36]. This dataset includes 40 cultivated rice accessions that together represent the major groups of Asian cultivated rice, and 10 wild rice samples - five accessions each from *Oryza rufipogon* and *O. nivara*. We aligned all reads from each accession onto the rice reference genome of Nipponbare (TIGR6.1) using BWA v0.5.8c [90, 91] with parameters ‘bwa aln -e 10’ and ‘bwa sampe -o 1000’. The alignment bam files were indexed and sorted with samtool v0.1.18 [92]. Read pair duplicates were removed using Picard (http://broadinstitute.github.io/picard).

### Generating the CNV discovery set

To discover CNVs in these accessions, we applied three method of PE,SR and RD. However, each of these approaches has limitations in terms of the size and type of CNVs detected [77]. For example, pair-end mapping cannot detect CNVs where the read pairs do not flank the CNV breakpoints. Split-read analysis is limited that both breakpoints of the CNV must be contained within a single read. The read-depth approach cannot infer the precise breakpoints of CNV calls. Thus, to obtain a full range of high-confidence CNVs, we integrated the results from three CNV discovery tools by three steps. First, we merged CNV calls supported by at least two methods for each sample, applying a stringent 50 % reciprocal overlap criterion. Second, to validate the accuracy of the CNV calls and refine imprecise breakpoints, local *de novo* assemblies were performed using Velvet [93] and the contigs were aligned to the reference genome by Exonerate [94] [77]. Third, we merged CNV calls in at least three accessions. To classify the ancestral states of CNVs, we compared the regions containing the variations with their orthologous regions in *O. glaberrima*. Core-orthologous gene pairs between *O. glaberrima* and Nipponbare were used to define orthologous blocks. CNV regions including 2 kbp flanking sequences were aligned with the corresponding orthologous sequences to deduce the likely ancestral state. If the CNV region was absent in *O. glaberrima*, the variation was defined as an insertion. If it was present in *O. glaberrima*, we defined it as a deletion.

### CNV validation

PCR validation was performed in five randomly selected rice accessions, along with Nipponbare. The primers were designed by Primer5 [95], and the PCR mix used 2X Power Taq PCR MasterMix (No: PR1701) from BioTeke Corporation (Beijing, China). The published data, including CNVs detected by read-depth based method in the same population [36], microarray-based study in the *japonica* and *indica* subspecies [34], and BAC-based report in rice and three of its closest relatives [35], were used for comparison with the CNV data generated in this project*.*

### Analysis of the functional impact of CNVs

Gene positions were obtained from the TIGR database (http://rice.plantbiology.msu.edu/). CNV genes were annotated by InterProScan to assign Gene Ontology annotations [96]. The Gene Ontology (GO) enrichment was calculated using a hypergeometric distribution statistical testing method with false discovery rate (FDR) correction [97]. The rice-specific CNV genes and conserved CNV genes across species were identified by homologous clustering of CNV genes in rice, *S. bicolor* [98], and *O. brachyantha* using Blast software. For validation of the functional genes affected by CNVs, the orthologous regions of CNVs in *O. nivara*, *O. barthii*, *O. glaberrima*, *O. glumaepatula*, and *O. meridionalis* were used for alignments. These genome sequences were provided by I-OMAP (the International *Oryza* Map Alignment Project); PCR validation was performed in 18 selected rice accessions. The visualization of alignments used the ACT v11.0.0 software.

### Analysis of CNV formation mechanisms

The co-linearity analysis of CNV genes compared to *O. glaberrima* was performed according to the previous described method [50]. CNV formation mechanisms were inferred using the Breakseq pipeline [47].

### Statistical analyses and figures

Statistical analyses were performed using the R v2.15.1 software [99]. Figures were generated using R v2.15.1, Circos v0.63 [100], and the Intergrative Genomics Viewer v2.1.28 [101]. The diagrams of alignments were pursued with series of custom Perl scripts.

### Availability of supporting data

The CNV data have been deposited into NCBI dbVar, with the submission number of nstd96.

## Additional files

Additional file 1: Table S1. An overview of CNV events in each rice accession was presented. For each CNV event, we listed detailed information on its position, length, ancestral state, formation mechanism, allele frequency, and overlapped gene ID. For those 90 CNV events validated by PCR experiments, the primer sequences and the templates used were included. CNVs overlapping with previously published data were also indicated. The presence of each CNV event in rice accessions was listed separately. The functional annotation information for each CNV gene was displayed in a separate excel sheet. (XLSX 1293 kb) Additional file 2: Table S2. Detail Gene ontology (GO) enrichment analysis of CNV genes. Genes were annotated by InterProScan to assign GO annotations. The enrichment of GO was calculated using a hypergeometirc distribution statistical testing method with false discovery rate (FDR) correction by the Benjamini and Hochberg method. (XLSX 108 kb) Additional file 3: Table S3. List of functional CNV genes validated by PCR experiments or alignments in the Oryza species (XLSX 108 kb) Additional file 4: Figure S1. Statistically over-represented gene ontology (GO) categories for CNV genes. (TIF 78 kb) Additional file 5: Table S4. Homology analysis of the conserved CNV genes in the Nipponbare genome (XLSX 150 kb) Additional file 6: Table S5. The formation mechanisms of non-co-linear CNV genes inferred by signatures of breakpoints (XLSX 11 kb) Additional file 7: Table S6. The proportion of different CNV formation mechanisms in the Nipponbare genome (XLSX 20 kb) Additional file 8: Table S7. The formation mechanisms of non-collinear CNV genes inferred by signatures of breakpoints (XLSX 11 kb) Additional file 9: Table S8. The proportion of different CNV formation mechanisms (XLSX 10 kb)

## Abbreviations

CNV: Copy number variation
CGH: Comparative genomic hybridization
NGS: Next-generation sequencing
NHR: Non-homologous recombination
NAHR: Non-allelic homologous recombination
NHEJ: Non-homologous end joining
MMEJ: microhomology-mediated end joining
DSB: DNA double-strand break
PE: Pair-end
RD: Read depth
SR: Split-read
SNP: Single nucleotide polymorphism
GO: Gene ontology
FDR: False discovery rate
I-OMAP: The International *Oryza* Map Alignment Project
LD: Long day
eQTL: Expression quantitative trait locus
GWAS: Genome-wide association study
