# Supplementary figures and images for: The impact and origin of copy number variations in the Oryza species
Source: BMC Genomics. 2016 Mar 29;17:261. doi: 10.1186/s12864-016-2589-2 (PMC4812662; doi:10.1186/s12864-016-2589-2)

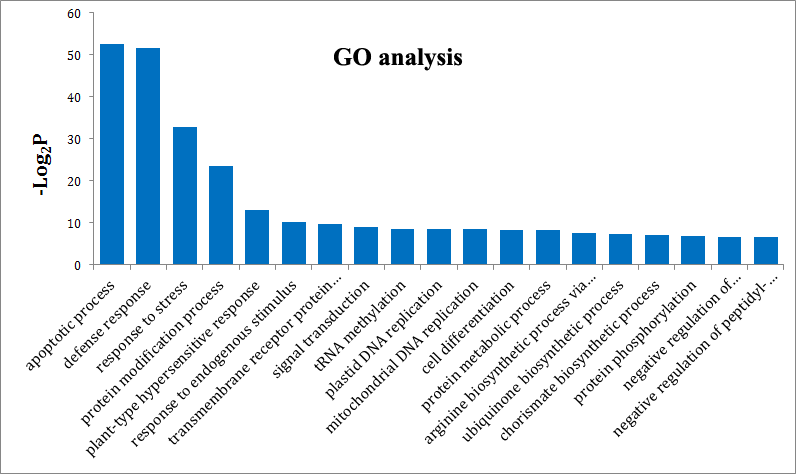

Supplement: Additional file 4: Figure S1. — Statistically over-represented gene ontology (GO) categories for CNV genes. (TIF 78 kb) [file 12864_2016_2589_MOESM4_ESM.tif]
